# Pseudouridylation-Related Genes Predict Prognosis and Therapeutic Response in Hepatocellular Carcinoma Patients

**DOI:** 10.7150/jca.117247

**Published:** 2025-08-16

**Authors:** Chenlu Lan, Donghua Gao, Yongguang Wei, Huasheng Huang, Xianwei Lv, Xin Zhou, Wei Qin, Xiwen Liao, Guangzhi Zhu, Tao Peng

**Affiliations:** 1 Department of Hepatobiliary Surgery, The First Affiliated Hospital of Guangxi Medical University, Nanning, Guangxi Zhuang Autonomous Region, People's Republic of China.; 2 Key Laboratory of High-Incidence-Tumor Prevention & Treatment (Guangxi Medical University), Ministry of Education, Nanning 530021, People's Republic of China.; 3 Guangxi Key Laboratory of Enhanced Recovery After Surgery for Gastrointestinal Cancer, 530021 Nanning, People's Republic of China.

**Keywords:** hepatocellular carcinoma, pseudouridylation, pseudouridine, molecular subtype, prognostic risk model, RNA modification

## Abstract

Emerging evidence has demonstrated that pseudouridylation regulates mRNA translation and gene expression, yet its molecular characteristics in hepatocellular carcinoma (HCC) remain unknown. Using public databases, we developed pseudouridylation-related molecular subtype and risk score model to assess HCC patient prognosis and disclose their clinical feature, molecular mechanism and immune landscape. Furthermore, quantitative polymerase chain reaction (qPCR) was performed to verify the expression of RDM1, CDCA3 and FLVCR1. Specifically, functional enrichment analysis revealed pseudouridylation-related genes (PRGs) predominantly regulate transcriptional and translational regulation. Prognostic PRGs stratified HCC into two distinct subtypes, the cluster 1 had a poor prognosis and was characterized by high alpha fetoprotein level, poor differentiation, advanced tumor stage, large tumor size, frequent TP53 mutation, up-regulation of cell cycle- and mitosis-associated genes, which was similar to the aggressive proliferation subtype of HCC. In contrast, the cluster 2 exhibited good prognosis and increased infiltration of immune cells, resembling the non-proliferation subtype of HCC, and suggesting its potential responsiveness to immunotherapy. Survival analysis discovered that the risk score model served as an independent prognostic factor, with high-risk group exhibiting significantly shorter overall survival and recurrence-free survival than low-risk group. Notably, receiver operating characteristic analysis revealed that the risk model had a powerful predictive performance for 1- and 3- year survival (AUC=0.806). In addition, functional enrichment analysis suggested that upregulated genes of high-risk group displayed an enrichment of cell cycle progression, mitotic division, and some oncogenic signaling pathways (PLK1, FOXM1, and p53 signaling pathways). qPCR experiment confirmed the significant overexpression of RDM1, CDCA3, and FLVCR1 in HCC tissues, being consistent with public database analysis. In conclusion, pseudouridylation related-molecular subtype and risk model may effectively predict the prognosis and therapeutic response of HCC.

## Introduction

Primary liver cancer is the fifth most common cancer and the second leading cause of cancer-related deaths, with its incidence and mortality ranking sixth and third among 36 cancers, respectively 1. Hepatocellular carcinoma (HCC) is the most common pathological type of liver cancer, accounting for 75%-85% of cases 2. Unfortunately, more than half of HCC patients are diagnosed at the middle or advanced stages during first visit 3, and the prognosis remains very poor with the 5-year survival rate being lower than 20% 4. In recent years, immunotherapy advances show a promising role on improving the survival of HCC 5. However, the heterogeneity of immunotherapy responses and the emergence of drug resistance pose major challenges that need to be overcome 6. Therefore, it is necessary to identify molecular subtype and develop new biomarkers for predicting the immunotherapy response and prognosis of HCC 7.

Advances in high-throughput sequencing technologies have enabled the detection of pseudouridine (Ψ) in human mRNA and are facilitating the elucidation of its biological functions. Ψ is a ubiquitous modified nucleotide and is dynamically regulated in human mRNA 8, 9. It has been reported that Ψ can affect pre-mRNA processing through pre-mRNA modification 10, suggesting its potential role on regulating gene expression. Current research on pseudouridylation in cancer is progressing, with findings demonstrating that pseudouridine modifications contribute to the progression of various cancers by regulating translation and gene expression 11. For instance, DKC1 overexpression impacts RNA pseudouridylation or telomerase activity, which promotes the synthesis of oncogenic proteins and drives tumor progression in gastric cancer 12, colorectal cancer 13 and uterine corpus endometrial carcinoma 14. RPUSD1 overexpression enhances eIF4E expression via its RluA catalytic domain, and activates the PI3K/AKT signaling pathway to promote malignant phenotypes of non-small cell lung cancer cells 15. PUS1 overexpression facilitates cell migration in clear cell renal cell carcinoma by promoting mRNA pseudouridylation and stabilizing SMOX gene transcripts 16. PUS7-dependent tRNA modifications regulate the growth and proliferation of colorectal cancer 17, pancreatic cancer 18 and gastric cancer cell 19 by modulating the translation of key genes. In HCC, PUS1-mediated pseudouridylation has been reported to enhance the translation of oncogenes, thereby promoting HCC progression 20. A study demonstrated PUS1 involves in HCC progression through regulating c-MYC and mTOR signaling pathways 21, consistent with our previous findings 22. However, research about pseudouridine synthases remains limited in HCC.

Therefore, it is meaningful and innovative to preliminarily explore the pseudouridylation-related transcriptomic features using bioinformatics approaches.

## Materials and Methods

### Data sources

**Figure 1:**
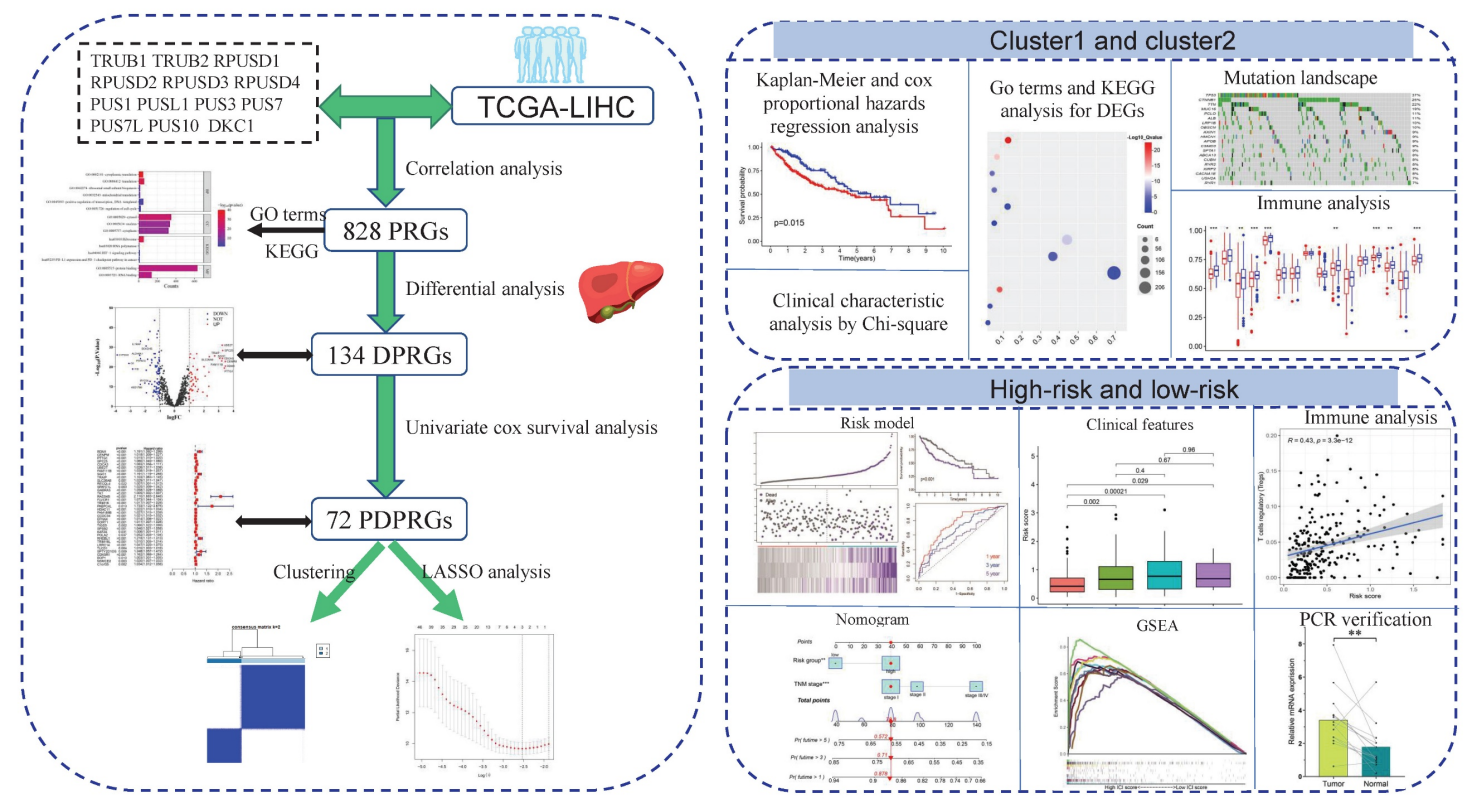
**The graphical abstract displayed the main methods and results of our study.** PRGs: pseudouridylation-related genes; DPRGs: Differential PRGs; PDPRGs: Prognostic DPRGs; DEGs: Differentially expressed genes; PCR: Polymerase chain reaction.

The methodological route of this study is summarized in Figure 1.

Genome expression profiles and clinical data of 370 tumor samples and 50 normal liver samples were download from The Cancer Genome Atlas (TCGA; https://cancergenome.nih.gov/) database to be a training cohort, samples with survival time shorter than 30 days were excluded. The International Cancer Genome Consortium (ICGC) cohort, containing 202 normal liver tissues and 240 HCC tissues, were downloaded from the ICGC portal to be the validation cohort. 75% patients of this cohort had chronic hepatitis B /C virus infection 23. Though the proportion of viral hepatitis-associated HCC cases in the TCGA cohort was limited, the two cohorts covered the majority of HCC populations with different etiologies.

Pseudoureoside synthase genes—including TRUB1, TRUB2, RPUSD1, RPUSD2, RPUSD3, RPUSD4, PUS1, PUSL1, PUS3, PUS7, PUS7L, PUS10 and DKC1—were identified through previous studies 22, 24.

### Correlation and differential analysis

To identify pseudouridylation-related genes (PRGs), a Pearson correlation analysis between 19937 protein-coding genes and 13 pseudoureside synthetase genes was performed using the normalized RNAseq data of TCGA cohort. Genes with p-value < 0.001 and correlation coefficient > 0.4 were defined as PRGs in this study. Subsequently, differential expression analysis between HCC and normal liver samples was conducted among these PRGs using the limma R package, genes exhibiting |log2(fold change)| >1 and adjusted p-value <0.05 were identified as differential PRGs (DPRGs).

### Identification of prognostic DPRGs and molecular subtypes

Univariate Cox survival analysis was performed to identify prognostic DPRGs (PDPRGs) using survival and survminer R packages. Subsequently, in order to investigate the molecular subtyping of PDPRGs, the expression data of these PDPRGs were applied for consistent clustering analysis by the ConsensusClusterPlus R package.

### Construction of risk score model and nomogram

Furthermore, least absolute shrinkage and selection operatorregression analysis was performed to reduce the collinearity of PDPRGs and build a prognostic risk score model using glmnet and survival R packages. The risk score was calculated using the following formula: risk score = (β₁ × expression₁) + (β₂ × expression₂) + … + (βₙ × expressionₙ), where β represents the survival coefficient for each gene. Subsequently, samples were divided into high- and low-risk groups based on the median risk score, and Kaplan-Meier survival analysis was used to analyze the prognostic significance of risk score groups. Finally, the predictive performance of the model was evaluated via receiver operating characteristic (ROC) analysis.

To investigate the clinical utility of risk score, a nomogram integrating risk score and TNM stage was developed using the rms R package. The predictive accuracy of the nomogram was validated through calibration curves, and ROC analysis was conducted to estimate the prognostic performance of nomogram, risk score and TNM stage, respectively.

### Survival and clinical characteristic analysis

Kaplan-Meier survival analysis was employed to screen the OS-related clinical variables (p<0.05). Multivariate Cox proportional hazards regression analysis was then used to assess the independent prognostic value of cluster groups and risk score groups.

Associations between cluster groups and clinical variables were examined using Chi-square test in R software. Additionally, differences of risk score across different clinical subgroups were analyzed using Wilcoxon test.

### Functional enrichment analysis

Database for Annotation, Visualization and Integrated Discovery (DAVID, https://david.ncifcrf.gov/) is bioinformatics platform that provides functional annotation tools for researchers to mine potential biological insights through uploading a gene list 25. Based on the differential expression analysis between cluster 1 (C1) and cluster 2 (C2), we submitted a list of differentially expressed genes (DEGs) on DAVID portal, and retrieved significantly enriched functional terms.

Gene Set Enrichment Analysis (GSEA) software (version 4.3.2) is a computational method to identify enriched gene sets associated with specific biological processes, pathways, or diseases using RNA-seq data and predefined gene set annotations. For both TCGA and ICGC cohorts, GSEA was performed by integrating RNA-seq expression profiles with sample risk group classifications, gene sets meeting the thresholds of p-value < 0.05 and discovery rate < 0.25 were defined as significantly upregulated or downregulated.

### Analysis of immune microenvironment, cell infiltration and functional states

The estimate R package utilized to calculate the tumor microenvironment (TME) score for each sample. The CIBERSORT algorithm was adopted to estimate immune cell abundances in R software, and the single-sample gene set enrichment analysis (ssGSEA) was employed to assess immune cell infiltration levels and immune-related functional activity with the GSVA and GSEABase R packages. Tumor Immune Dysfunction and Exclusion (TIDE) scores were obtained from the TIDE portal (http://tide.dfci.harvard.edu/) to predict the immune response in HCC. All the immune evaluation algorithms were implemented based on gene expression profiles of TCGA and ICGC cohorts.

Subsequently, differences of TME score, ssGSEA score, TIDE score, and immune checkpoint gene expression levels across cluster subgroups and risk subgroups were analyzed using the Wilcoxon test. Spearman correlation analysis was conducted to evaluate the association between risk scores and immune cell abundances.

### Mutation analysis

Genome-wide somatic mutation data of TCGA cohort was downloaded from the Genomic Data Commons portal (https://portal.gdc.cancer.gov/). The maftools R package was utilized to visualize and analyze the somatic mutation landscape of molecular subtypes C1 and C2.

### Drug sensitivity prediction analysis

Drug sensitivity analysis was performed using the oncoPredict R package. The sensitivity (IC50 values) of 367 drugs in the GDSC1 database was predicted based on mRNA expression data of RDM1, CDCA3 and FLVCR1. Differences between IC50 values and risk groups were statistically compared using the Wilcoxon test.

### Verification of expression and prognostic significance of PDPRGs

The Gene Expression Profiling Interactive Analysis (GEPIA; http://gepia.cancer-pku.cn/) platform was utilized to assess the differential expression of RDM1, CDCA3 and FLVCR1 between normal liver tissues and HCC tissues, as well as across different tumor stages. The Kaplan-Meier Plotter database (https://www.kmplot.com/analysis/) was employed to evaluate the prognostic significance of these genes in terms of overall survival.

### Quantitative polymerase chain reaction (qPCR)

Twenty paired HCC and adjacent non-tumor liver tissue samples were collected from the First Affiliated Hospital of Guangxi Medical University. Total RNA was isolated from tissues using TrizolTM reagent (Invitrogen, USA), and cDNA synthesis was performed with the PrimeScript™ RT reagent kit (Takara, Japan). qPCR was completed using the FastStart Universal SYBR® Green Master Mix (Roche, Germany), Relative mRNA expression levels were calculated via the 2-∆∆CT method, with primer sequences listed in Table 1.

All specimens were derived from HBV-associated HCC patients with BCLC stage A or B, and were histologically confirmed as HCC by postoperative pathology. Written informed consent was obtained from all patients, and this study was approved by the Ethical Review Committee of the First Affiliated Hospital of Guangxi Medical University [Approval Number: 2025-E0484].

### Statistical analysis

Kaplan-Meier survival analysis and Cox proportional hazards regression analysis were conducted using SPSS 22.0 software. Hazard ratios (HR) and 95% confidence intervals were calculated to quantify prognostic risks. Pearman correlation analysis, differential expression analysis, univariate Cox survival analysis and Wilcoxon tests were performed in R software (version 4.3.2). Paired Student's t-test was employed for the statistical analysis of PCR experiment. A threshold of p < 0.05 was considered statistically significant.

## Results

### Identification of prognostic DPRGs in the TCGA cohort

**Figure 2:**
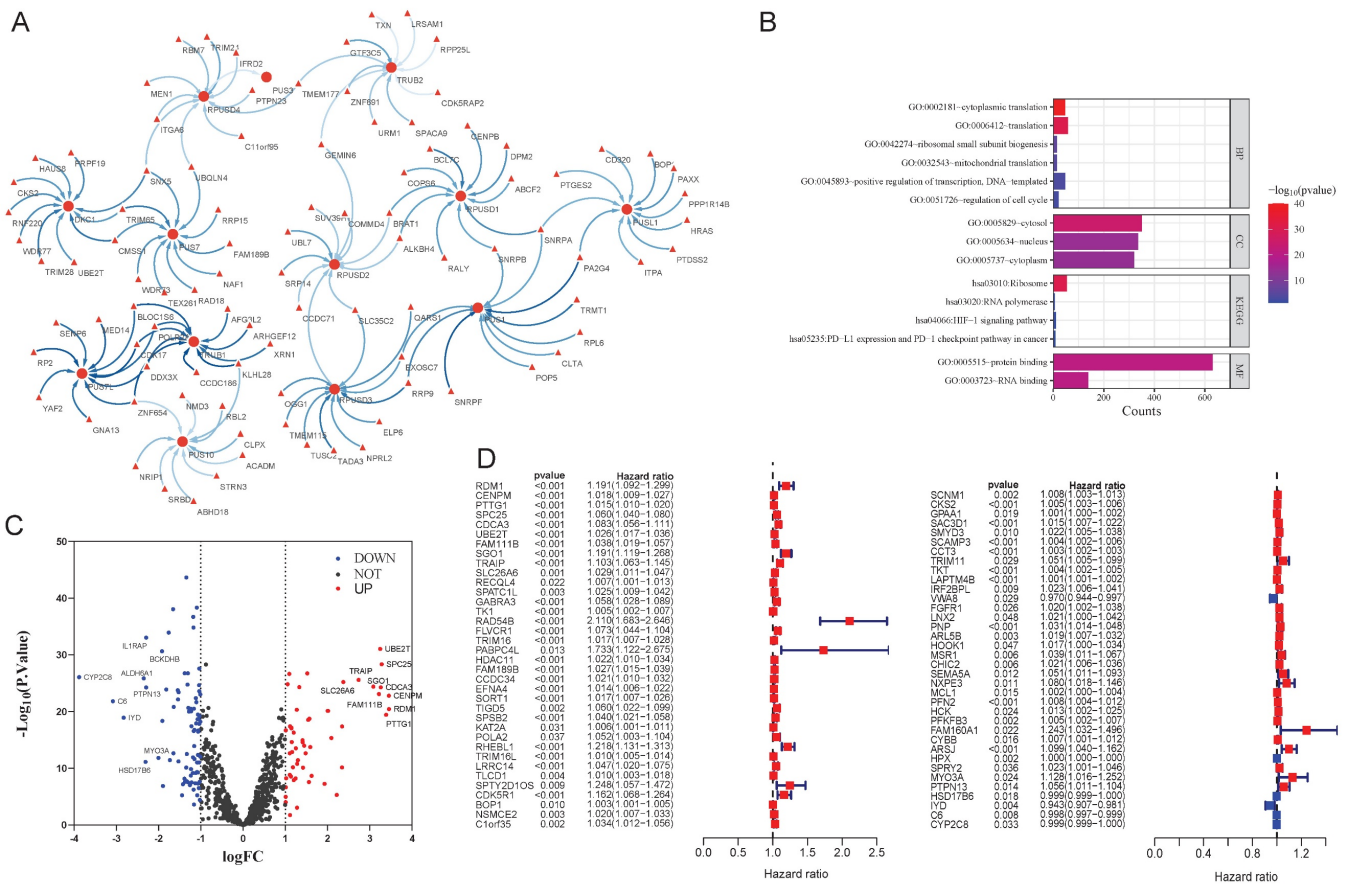
** Identification of prognostic and differential pseudouridylation-related genes (PRGs). A**: Co-expression network of top 100 genes ranked with correlation coefficients. **B**: Gene ontology terms and KEGG pathways of PRGs. **C**: Differential expression analysis for PRGs between HCC and normal liver tissues. **D**: Univariate cox survival analysis of differential PRGs.

Through correlation analysis in the TCGA cohort, 828 PRGs were identified (Table S1). The co-expression network visualized the top 100 most strongly correlated genes (Figure 2A). Functional enrichment analysis suggested that these PRGs were significantly enriched in translation, ribosomal small subunit biogenesis, mitochondrial translation, cell cycle and PD-1 checkpoint pathway (Figure 2B). Differential expression analysis identified 54 upregulated and 82 downregulated PRGs in HCC tissues compared to normal liver tissues (Figure 2C, Table S2). Subsequent univariate Cox survival analysis of these 134 PRGs determined 72 genes significantly associated with OS (Figure 2D).

### Development of prognostic DPRGs-related molecular subtype

**Figure 3:**
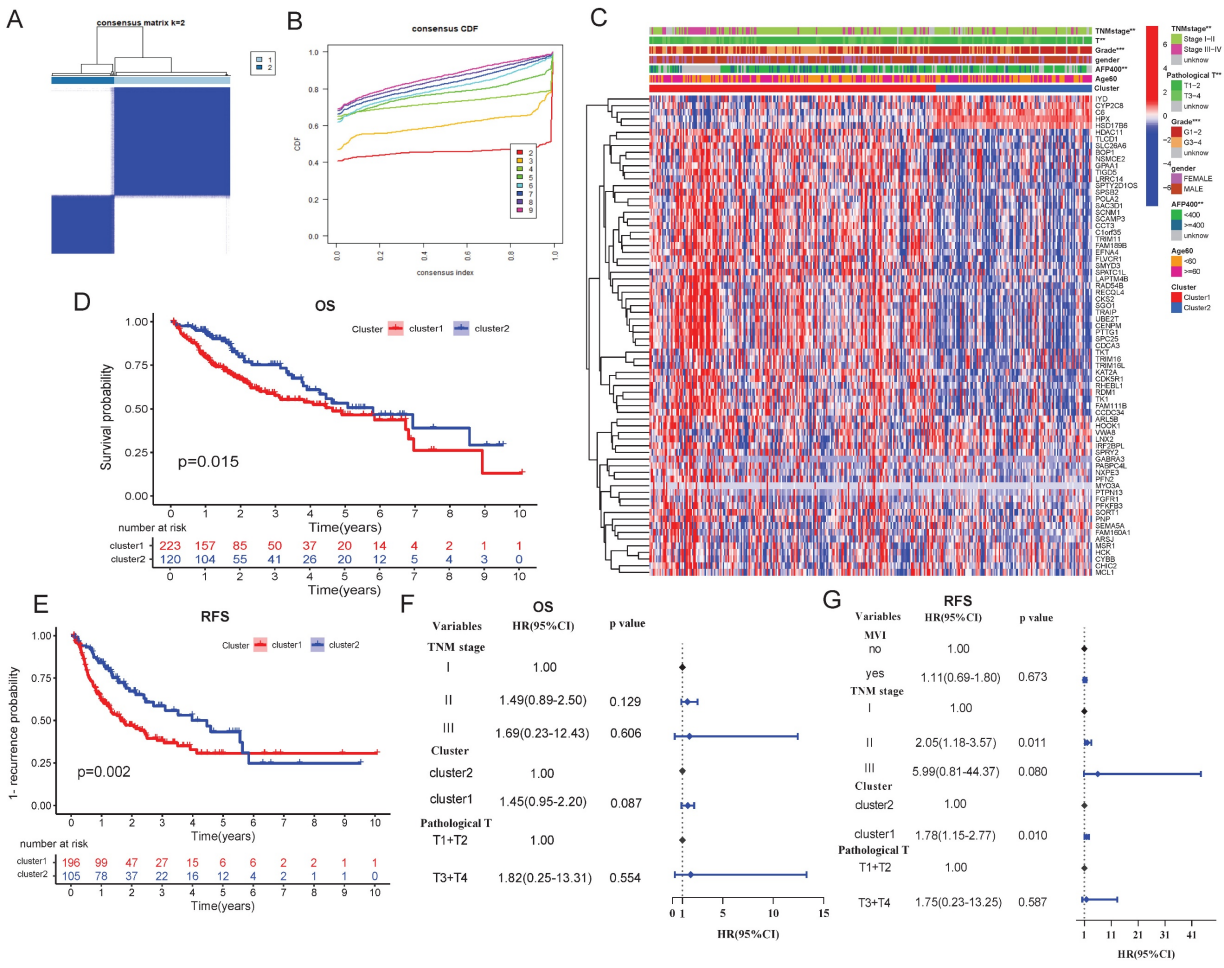
** Consistency cluster analysis. A**: Consistency clustering divided TCGA-LIHC samples into two clusters. **B**: Consensus clustering cumulative distribution functions for k = 2 to 9. **C**: Correlation heatmap between clinicopathologic features and clusters. **D-E**: Kaplan-Meier analysis of clusters for OS (**D**) and RFS (**E**). **F-G**: Multivariate cox proportional hazards regression model of clusters for OS (**F**) and RFS (**G**).

Consistent clustering analysis stratified HCC samples into two distinct molecular subtypes (C1 and C2) with a ratio of 1:1.9 (Figure 3A-B). Compared to C2, the C1 subtype exhibited significantly higher serum alpha-fetoprotein (AFP) levels, poorer pathological grade, larger tumor size, and more advanced TNM stage (Figure 3C). Survival analysis demonstrated that the C1 had markedly shorter OS (Figure 3D) and recurrence-free survival (RFS; Figure 3E). Multivariate Cox regression confirmed the C1/C2 classification as an independent prognostic factor (Figure 3F-G).

**Figure 4:**
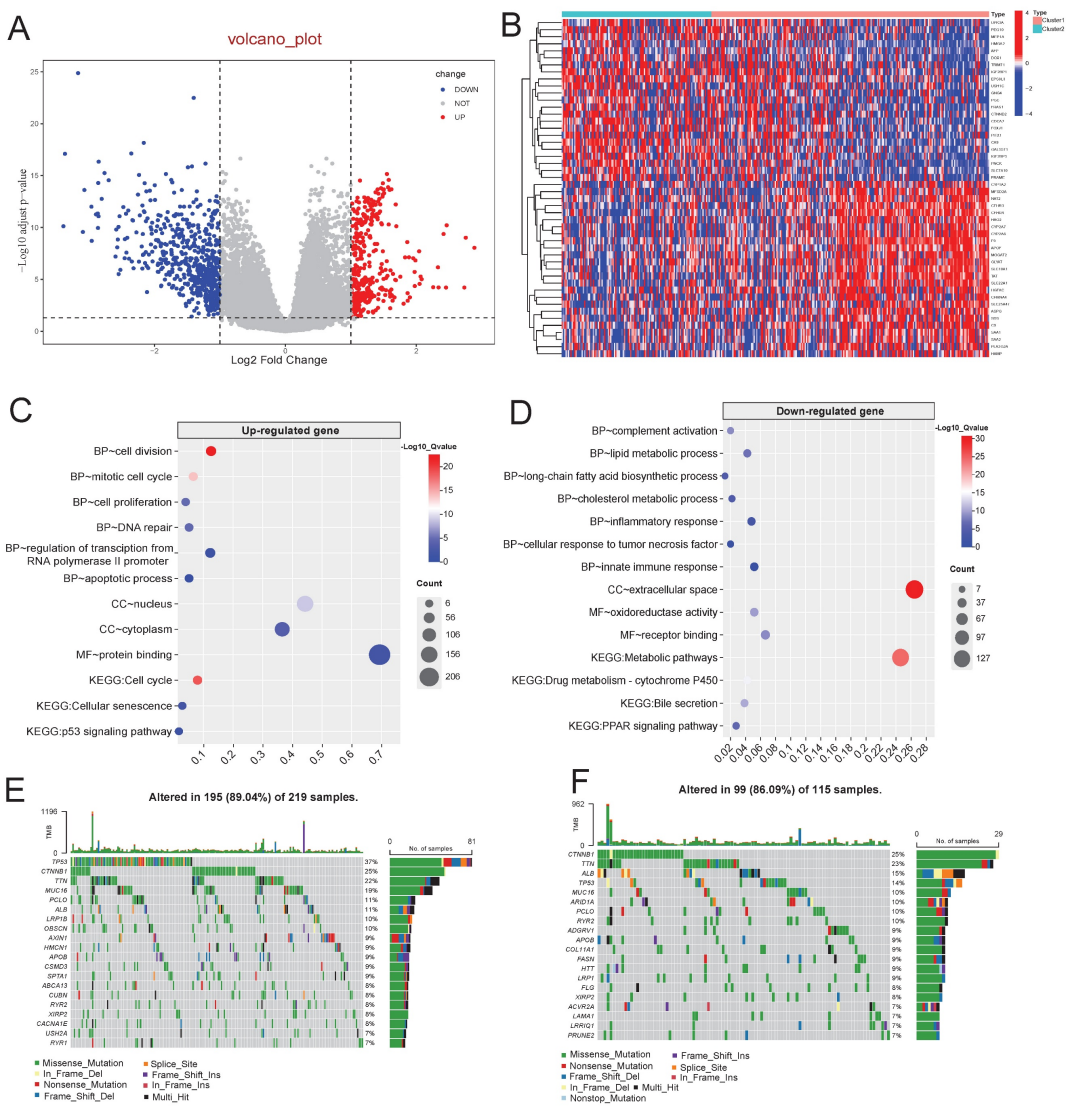
**Molecular mechanism of different clusters. A**: Differential expression analysis between cluster1 and cluster2. **B**: Heatmap of top 25 up-regulated genes and down-regulated genes. **C-D**: Functional enrichment analysis of up-regulated genes (**C**) and down-regulated genes (**D**). E-F: Mutation landscape of cluster1 (**E**) and cluster2 (**F**).

To investigate the underlying molecular mechanism behind these two clusters, 900 DEGs were identified between C1 and C2 (Figure 4A-B, Table S3) and subjected to functional enrichment analysis via DAVID platform. Upregulated DEGs in C1 were significantly enriched in cell cycle, cell proliferation, cell division, apoptotic process and p53 signaling pathway (Figure 4C). Conversely, downregulated DEGs in C1 showed associations with inflammatory response, cellular response to tumor necrosis factor, metabolism and PPAR signaling pathway (Figure 4D).

### The differences of TP53 mutation and immune landscapes between two molecular subtypes

**Figure 5:**
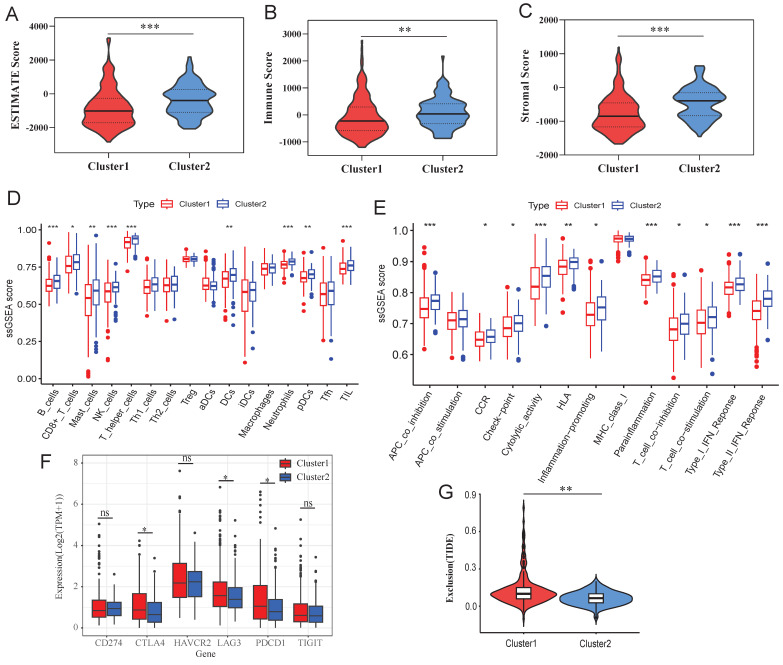
** Immune analysis of different clusters. A-C**: Differences of estimate score (**A**), immune score (**B**) and stromal score (**C**) between different clusters. **D**: Differences of immune cell infiltration between different clusters. **E**: Differences of immune function between different clusters. **F**: Expression differences of immune checkpoint genes between different clusters. **G**: Difference of exclusion score between different clusters. *: p<0.05, **: p<0.01, ***: p<0.001, ns: no significance.

To validate the dysfunction of p53 signaling and inflammatory pathways, we analyzed genomic mutations and immune landscapes, and found that C1 subtype exhibited a significantly higher TP53 mutation rate than C2 (37% vs. 14%; Figure 4E-F), aligning with the functional enrichment analysis above. Immune analysis discovered that C2 appeared higher estimate, immune and stromal score compared to C1 (Figure 5A-C), indicating lower immune infiltration level in C1 subtype.

In addition, ssGSEA further demonstrated lower of cytotoxic immune cells in C1, including B cells, CD8+ T cells, mast cells, NK cells and DCs (Figure 5D). C1 subtype also showed suppressed immune-related functional activity, with diminished checkpoint, cytolysis, inflammatory and IFN responses (Figure 5E), suggesting the C1 subtype exhibits an immune-cold phenotype. Moreover, some immune checkpoint genes, such as CTLA4, LA3 and PCCD1, were overexpressed in C1 (Figure 5F), implying C1 subtype may have occurred immunosuppression. TIDE analysis confirmed higher immune exclusion score in C1 (Figure 5G), explaining that immune cells exclusion may be the cause of immune-cold phenotype for C1.

### Prognostic DPRGs-based risk score model

**Figure 6:**
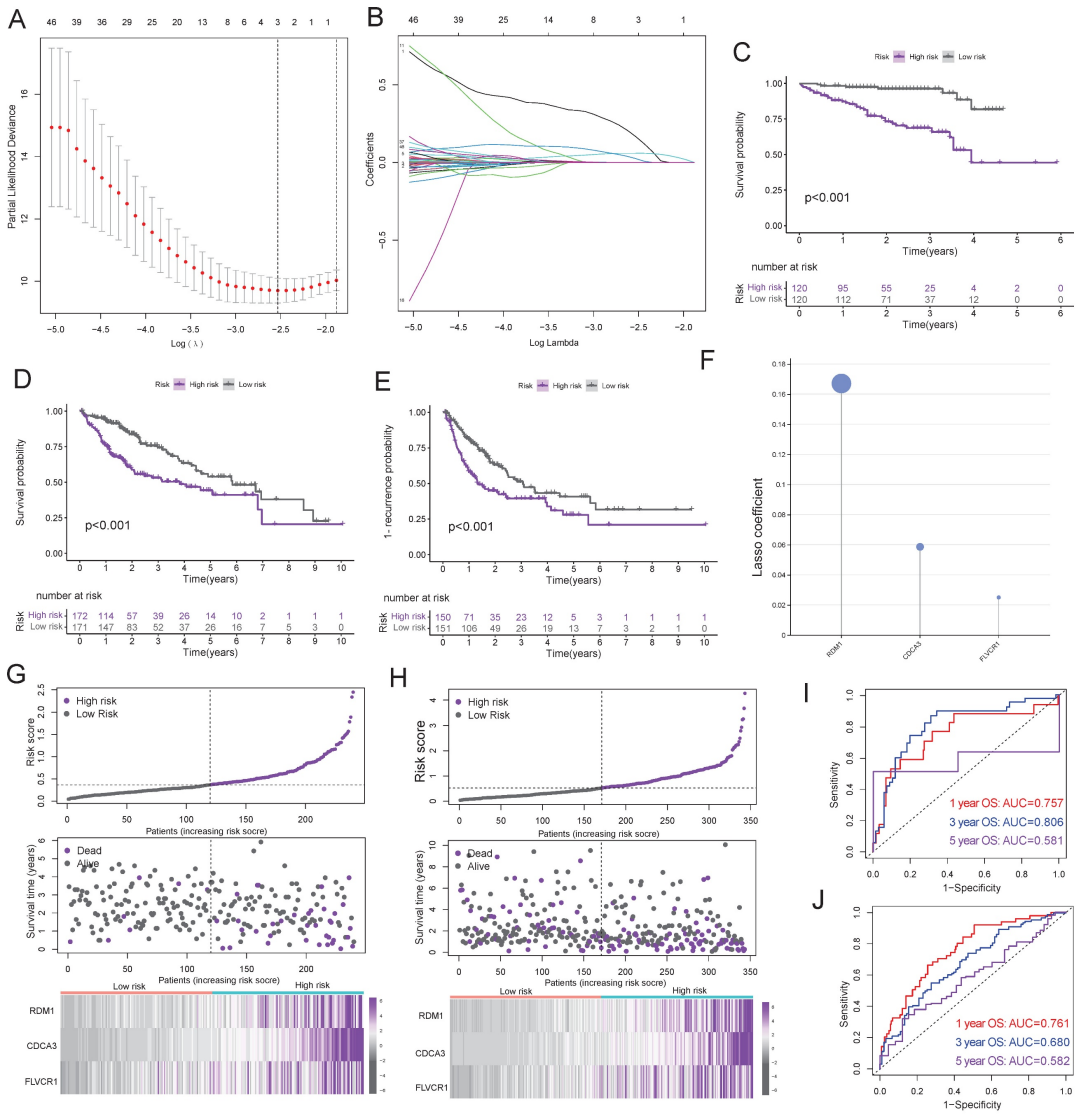
** Identification and validation of risk score signature. A**: Cross-validation of PDPRGs in the LASSO regression. **B**: LASSO coefficients of PDPRGs. **C-D**: Survival curves of risk groups in ICGC (**C**) and TCGA (**D**) cohorts. **E**: Recurrence curves of risk groups in TCGA cohort. **F**: LASSO regression coefficient of RDM1, CDCA3 and FLVCR1. **G-H**: Risk scores, survival status and gene expression heatmap of ICGC (**G**) and TCGA (**H**) cohorts. **I-J**: Prognostic ROC of risk score in ICGC (**I**) and TCGA (**J**) cohorts.

RDM1, CDCA3 and FLVCR1 were selected through LASSO regression analysis to construct the risk score model (Figure 6A-B). Using the median risk score as the cutoff, TCGA and ICGC cohorts were stratified into high-risk and low-risk groups, Kaplan-Meier survival analysis demonstrated high-risk group significantly suffered worse OS and RFS than low-risk group (Figure 6C-E). The survival coefficients for RDM1, CDCA3, and FLVCR1 were 0.167, 0.058, and 0.025, respectively (Figure 6F). Scatter plots showed HCC mortality rates escalated with increasing risk scores, while prolonged survival times were observed in low-risk groups (Figure 6G-H). ROC curves showed robust predictive performance, with AUC 1-/3-/5-year AUC values of 0.757/0.806/0.581 (ICGC, Figure 6I) and 0.761/0.680/0.582 (TCGA, Figure 6J), indicating superior accuracy for short-to-medium term survival prediction (1-3 years).

### The predictive power of nomogram was stronger than TNM stage

**Figure 7:**
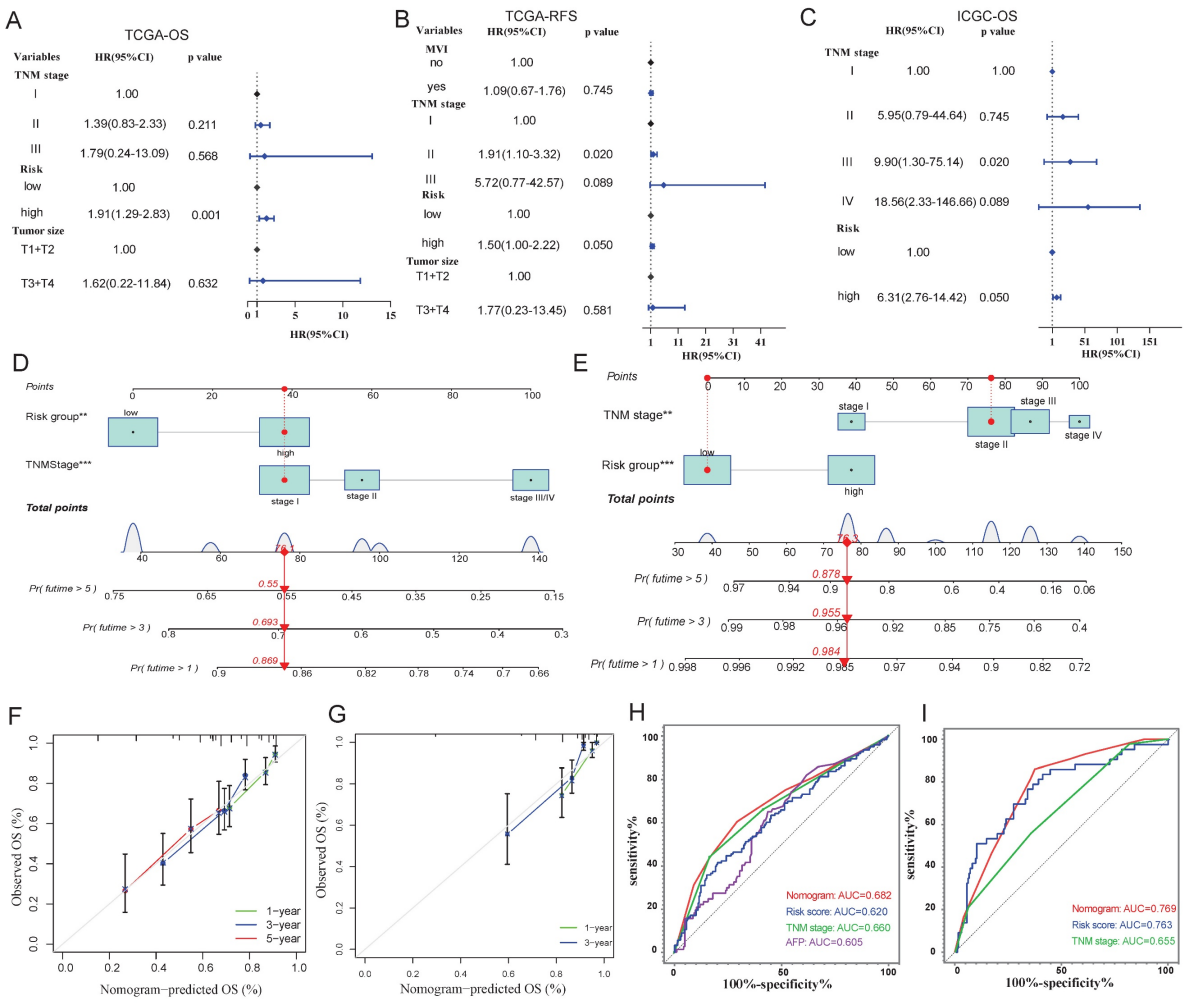
**Construction of cox proportional hazards regression model and nomogram. A-C**: Multivariate cox proportional hazards regression models in TCGA (**A** and **B**) and ICGC (**C**) cohorts. **D-E**: Nomograms of TCGA (**D**) and ICGC (**E**) cohorts. **F-G**: Calibration curves of nomogram in TCGA (**F**) and ICGC (**G**) cohorts. **H-I**: Prognostic ROC of nomograms, risk score and clinical variables in TCGA (**H**) and ICGC (**I**) cohorts.

Kaplan-Meier survival analysis identified TNM stage, tumor size and microvascular invasion as significant prognostic variables (Table 2-4). Multivariate Cox proportional hazards regression analysis incorporating these clinical factors confirmed the risk-score group as an independent prognostic predictor (Figure 7A-C).

The nomograms composed of TNM stage and risk group (Figure 7D-E) were proved to have a good predictive performance by calibration plots (Figure 7F-G). Furthermore, ROC curves indicated that the predictive power of nomogram was better than TNM stage (Figure 7H), and predictive ability of risk score and nomogram were both better than TNM stage in ICGC cohort (Figure 7I).

### The clinical characteristic of risk score and stratified survival analysis

**Figure 8:**
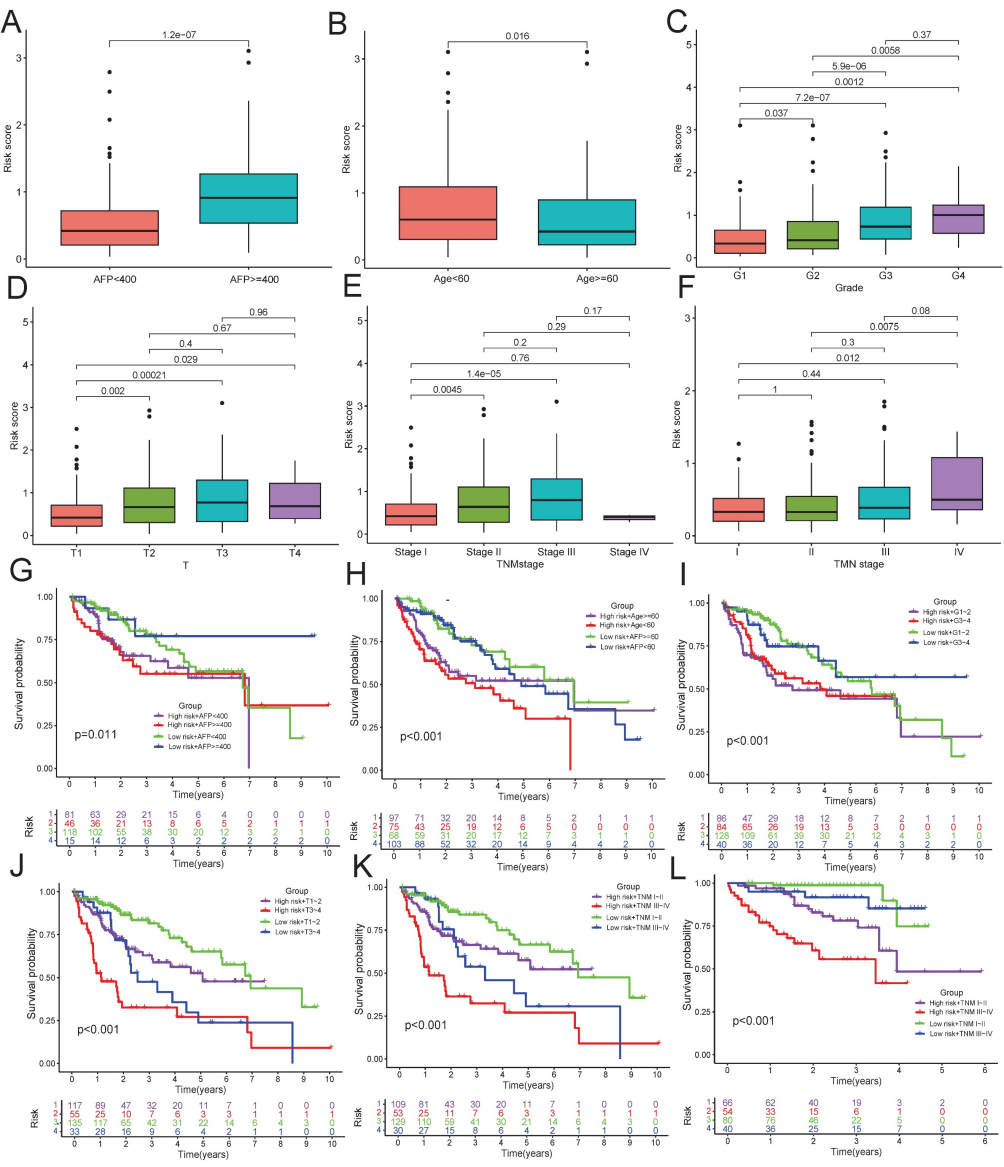
**The difference and prognosis of risk score in different clinical feature. A-F**: The differences of risk score were compared in AFP (**A**), age (**B**), pathological grade (**C**), tumor size (**D**) and TNM stage subgroups (**E**: TCGA, **F**: ICGC). **G-L**: Survival analysis of risk group combined with AFP (**G**), age (**H**), pathological grade (**I**), tumor size (**J**) and TNM stage subgroups (**K**: TCGA, **L**: ICGC).

Clinical characteristic analyses showed that elevated risk scores were significantly associated with adverse clinicopathological features, including AFP >= 400 ng/ml, age<60 years, poor pathological grade, larger tumor size and advanced TNM stage (Figure 8A-F). To delineate synergistic prognostic effects, patients were stratified into four subgroups by integrating risk group with dichotomized clinical variables. Univariate survival analysis manifested high-risk patients with AFP >= 400 ng/ml, age<60 years, grade G3/G4 or TNM stage III/IV had the worst poorest survival outcomes (Figure 8G-L).

### Functional enrichment analysis of risk group

**Figure 9:**
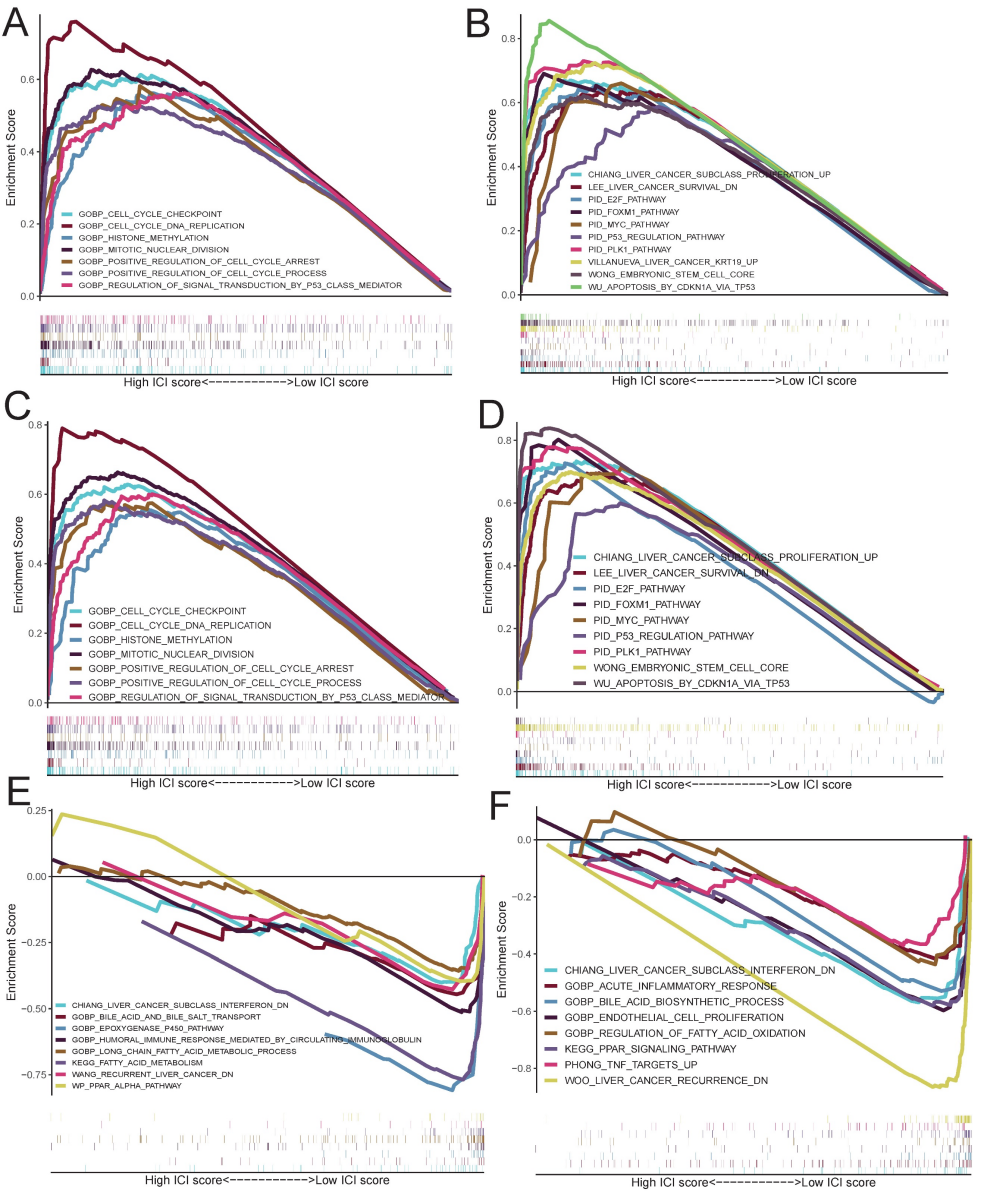
** Functional enrichment analysis of risk group. A-B: Upregulated gene sets of high-risk group in TCGA cohort based on the official c5.** (**A**) and c2 (**B**) files. **C-D**: Upregulated gene sets of high-risk group in ICGC cohort based on the official c5 (**C**) and c2 (**D**) files. **E-F**: Downregulated gene sets of high-risk group in TCGA (**E**) and ICGC (**F**) cohorts.

GSEA of the TCGA cohort identified significant upregulation of some gene sets in high-risk group, including positive regulation of cell cycle process, mitotic nuclear division, regulation of signal transduction by p53 class mediator, positive regulation of cell cycle arrest and methylation (Figure 9A). Furthermore, KEGG pathway analysis further revealed significant enrichment of oncogenic pathways in high-risk group, such as PLK1 pathway, FOXM1 pathway, E2F pathway, MYC pathway and p53 regulation pathway (Figure 9B). Particularly, these pro-tumorigenic enriched gene sets were verified in the ICGC cohort (Figure 9C, D). In low-risk group, some metabolic and immune-related gene sets were significantly enriched, which be same with the C2 subtype (Figure 9E, F).

### Immune-related and drug sensitivity analysis

**Figure 10:**
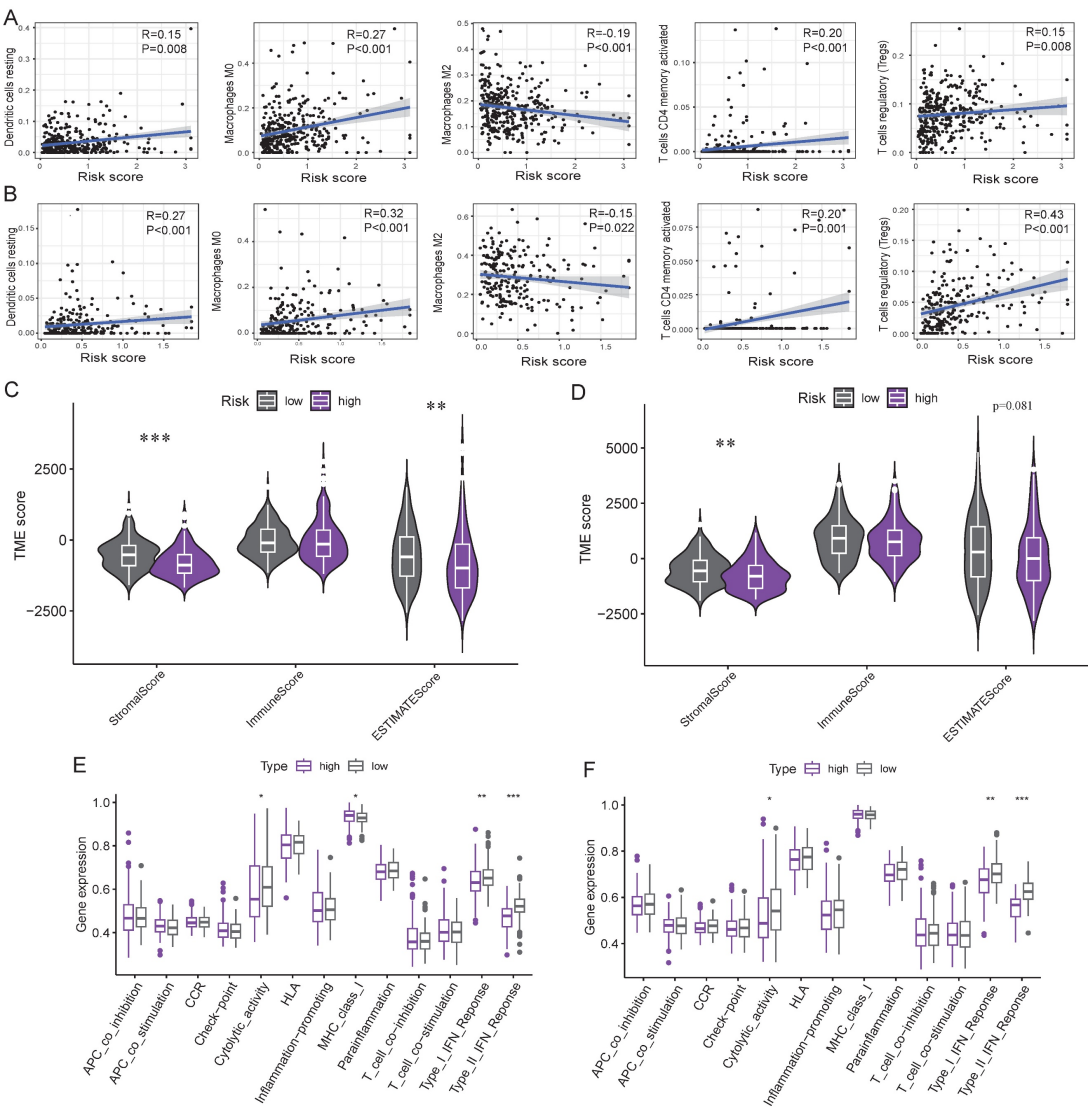
** Immune-related analyses. A-B**: Correlation of risk score and immune cells in TCGA (**A**) and ICGC (**B**) cohorts. **C-D**: Differences of TME score in TCGA (**C**) and ICGC (**D**) cohorts. **E-F**: Differences of immune function in TCGA (**E**) and ICGC (**F**) cohorts. *: p<0.05, **: p<0.01, ***: p<0.001.

Correlation analysis revealed risk score was positively related to the infiltration of macrophages M0 cells, dendritic cells, T cells CD4 memory activated and T cells regulatory (Tregs) cells, but negatively related to macrophages M2 cells (Figure 10A-B). Low-risk group exhibited a higher stromal and estimate score compared to high-risk group (Figure 10C-D). Furthermore, ssGSEA analysis displayed type I IFN response, type II IFN response, and cytolytic activity were more active in low-risk group (Figure 10E-F).

**Figure 11:**
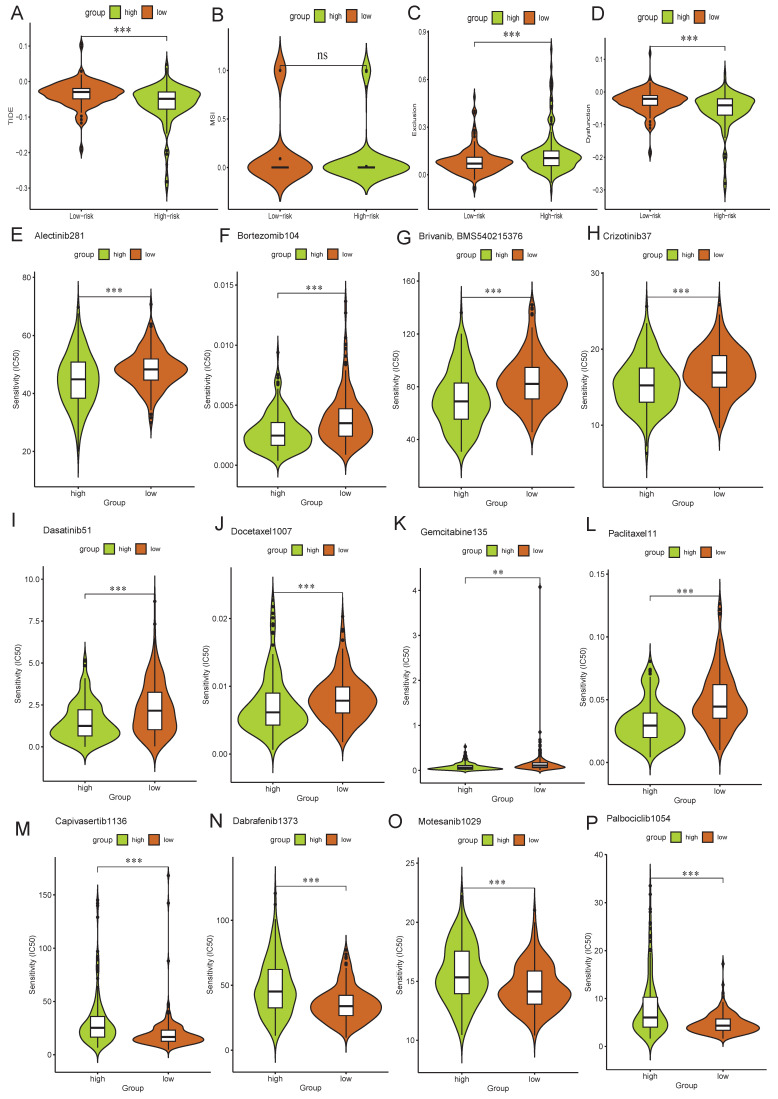
** Immune-related analyses. A-D**: The scores of TIDE (**A**), MSI (**B**), immune immune exclusion (**C**), and dysfunction (**D**) were compared between the high- and low-risk groups. **E-P**: Drug sensitivity analysis identified preferential drugs for high-risk group (**E-L**) and low-risk group (**M-P**). **: p<0.01, ***: p<0.001, ns: no significance.

TIDE analysis revealed that the high-risk group exhibited significantly lower TIDE and immune dysfunction scores, yet higher immune exclusion scores compared to the low-risk group (Figure 11A-D), suggesting diminished immunotherapeutic responsiveness in high-risk patients. Furthermore, drug sensitivity analysis demonstrated lower IC50 values for alectinib, bortezomib, brivanib, crizotinib, dasatinib, docetaxel, gemcitabine and paclitaxel in the high-risk group (Figure 11E-L), indicating enhanced therapeutic response to these drugs. Conversely, low-risk group displayed preferential sensitivity to capivasertib, dabrafenib, motesanib and palbociclib with lower IC50 values (Figure 11M-P).

### Verification of PDPRGs overexpression and prognostic significance in HCC

**Figure 12:**
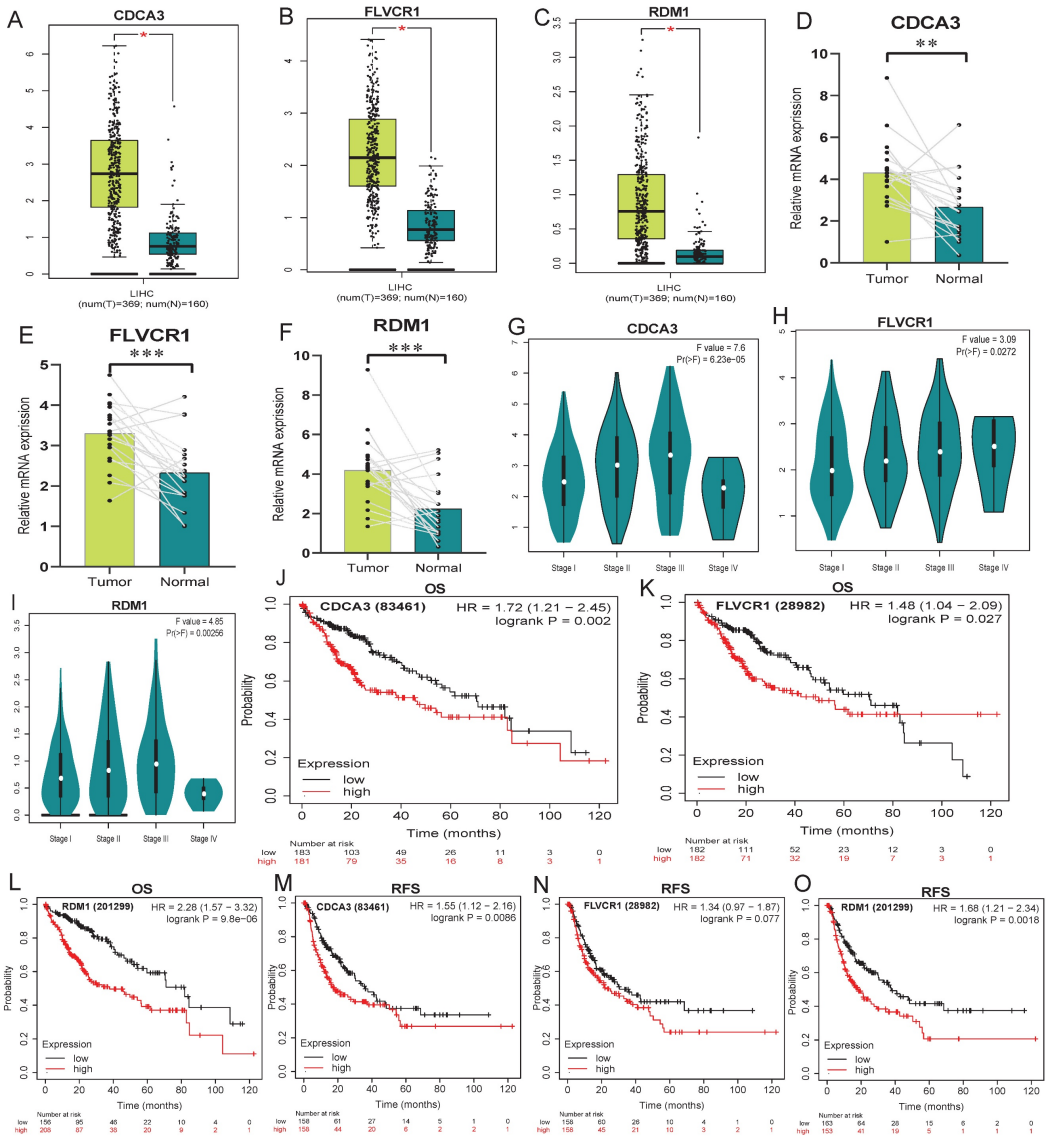
**The expression and prognostic value of RDM1, CDCA3 and FLVCR1. A-C**: Differential expression analysis in GEPIA platform. **D-F**: Results of PCR experiments. **G-I**: Differences of gene expression in tumor stage. **J-L**: K-M survival analyses for OS. **M-O**: K-M survival analyses for RFS. *: p<0.05, **: p<0.01, ***: p<0.001.

Results of GEPIA and PCR experiment confirmed that RDM1, CDCA3 and FLVCR1 were significantly upregulated in HCC tissues compared to normal liver tissues (Figure 12A-F). Similarly, there were significant expression differences for RDM1, CDCA3 and FLVCR1 within different tumor stages. Patients with stage III emerged a higher expression of RDM1, CDCA3 and FLVCR1 than stage I and stage II (Figure 12G-I). Kaplan-Meier analysis demonstrated overexpression of RDM1, CDCA3 and FLVCR1 had significantly worse OS and RFS compared to low-expression group (Figure 12J-O).

## Discussion

Genes usually function together within biological networks to execute complex cellular processes 26. Using genome-wide correlation analysis of 13 pseudouridine synthase genes, we identified 828 PRGs. Functional enrichment analysis revealed these PRGs mainly participate in translation, positive regulation of transcription, RNA polymerase, the activity of protein and RNA binding, suggesting PRGs may affect gene expression and protein expression in HCC. Besides, we discovered PRGs enriched in cell cycle, HIF-1 signaling pathway, PD-L1 expression and PD-1 checkpoint pathway, implying PRGs may affect cell growth, anaerobic metabolism and immune function in HCC 27-29.

Among these PRGs, differential analysis and survival analyses identified 72 core PDPRGs. Consensus clustering analysis of these PDPRGs stratified HCC into C1 and C2 subtypes.

Survival analysis revealed that C1 had significantly shorter OS and RFS than the C2. Correlation analysis between clinical variables and molecular clusters indicated that C1 was predominantly associated with advanced TNM stage, higher AFP, poor pathological grade, and larger tumor size, suggesting its more malignant characteristics. Functional enrichment analysis demonstrated upregulated genes of C1 involved in p53 signaling pathway, cell cycle, cell proliferation, apoptotic process and cell division. As previously reported, dysfunction of the p53 pathway promotes tumor progression by disrupting cell cycle, apoptosis, and proliferation 30, 31. The mutation analysis confirmed a higher TP53 mutation frequency in C1, implying p53 signaling pathway may play a role in C1. It is well known that the molecular classification of HCC is widely recognized to two mainly subtypes: the proliferation and non-proliferation subtypes 32, 33. The proliferation subtype, characterized by high AFP levels, poor differentiation, frequent TP53 mutations, up-regulation of cell cycle and mitosis, vascular invasion and unfavorable prognosis 34, closely aligns with the molecular features of C1 in our study.

In contrast, we found that the C2 subtype characterized by favorable clinical features and better prognosis, and exhibited the molecular signatures which metabolic and immune processes-related genes were distinctly upregulated. Specifically, these included metabolic pathways, bile secretion, oxidoreductase activity, PPAR signaling pathway, inflammatory response and response to tumor necrosis factor. The PPAR signaling pathway is known to regulate metabolic homeostasis and inflammatory responses in HCC 35, 36, and modulates immune cell function and infiltration by influencing metabolic reprogramming, oxidative stress, and inflammatory cytokine production 37. Through a series of immune-related analysis, our study revealed that the C2 emerged a higher immune cell infiltration and more active of immune function. These findings align with the reported molecular signatures of non-proliferation subtype, which characterized by metabolic reprogramming, positive prognosis, and enhanced immune microenvironment activity 34.

Furthermore, we developed a prognostic risk score signature, included RDM1, CDCA3 and FLVCR1, to predict clinical outcomes in HCC. Multivariate Cox regression analysis confirmed this signature as an independent prognostic factor. Notably, the nomogram model integrating with risk score and TNM stage achieved decent predictive performance with higher specificity and sensitivity than TNM stage alone, suggesting its potential as a clinical tool for predicting HCC prognosis.

Regarding the molecular features of high-risk and low-risk groups, we observed that elevated risk scores were predominantly observed in HCC patients with age<60 years, advanced TNM stage, poor pathological grade, larger tumor size or AFP>=400 ng/ml. Survival analysis further confirmed a significantly poorer prognosis when high-risk patients equipped with one of above-mentioned clinical features. From a molecular mechanistic perspective, GSEA showed that gene expression levels of cell cycle, mitotic division-related biological processes and several cancer-related pathways remarkably increased in high-risk group. Notably, dysregulation of PLK1 and MYC pathways was previously reported to drive uncontrolled proliferation and hepatocarcinogenesis 38, 39. Up-regulation of E2F and FOXM1 pathways were reported to promote cell proliferation and restrain apoptosis 40, 41. These results implied that high-risk group may regulate cell cycle, mitotic division, cell proliferation to accelerate HCC development. Indeed, available literatures had demonstrated CDCA3 overexpression was associated with poor prognosis of HCC and enhanced the migration and invasion of HCC cell via E2F pathway activation 42, 43. RDM1 and FLVCR1 overexpression was found to be correlated with poor OS and PFS in HCC 44, 45, though their functional roles in HCC remain unclear.

Tumor progression is closely associated with the dynamic composition of stromal cells, tumor cells and immune cells within the TME 46. Among these components, immune infiltration critically influences immune surveillance and anti-tumor responses 47. Traditionally, high immune infiltration coupled with favorable prognosis are classified as the "hot" immune subtype 48. This subtype is more likely to benefit from immunotherapy 49, particularly in cases with abundant CD8+ T cell infiltration 50. Through immune-related analyses, we observed that C2 displayed significantly higher immune cell infiltration compared to C1, including CD8+ T cells, mast cells, NK cells, B cells and DCs. Additionally, C2 exhibited higher activity scores of immune checkpoints, cytolysis, inflammation and IFN response, suggesting greater potential sensitivity to immune checkpoint inhibitors in C2 subgroup. Conversely, C1 demonstrated lower immune infiltration alongside upregulated immune checkpoint gene expression, which is an immunosuppressive TME type characterized by immune exhaustion 51, 52. Interestingly, no marked differences in overall immune infiltration were detected between risk groups. The low-risk group showed significantly stronger cytolytic activity and type I/II IFN responses compared to the high-risk group, implying more robust innate immune activation 53. Furthermore, the risk score positively correlated with dendritic cell density, M0 macrophage infiltration, activated CD4+ memory T cells, and Tregs cells, but inversely associated with M2 macrophage abundance. Given that Tregs cells suppress effector T cell function 54 and resting dendritic cells or M0 macrophages may facilitate immune evasion 55. These findings nearly align with the aggressive clinical phenotype observed in C1.

To our knowledge, this study provides the first systematic characterization of the molecular profiles and prognostic value of PDPRGs in HCC. However, several limitations should be acknowledged. First, the mechanisms of pseudouridylation remain poorly characterized due to limited research in cancer, a gap that will be the focus of our subsequent investigations. Furthermore, while our findings were derived from robust bioinformatics analyses, the absence of experimental validation represents a critical limitation that warrants resolution in future studies.

## Supplementary Material

Supplementary figures and tables.

## Figures and Tables

**Table 1:** Primer sequences for PCR.

Gene	Primer sequences	
GAPDH	forward	GTCAGCCGCATCTTCTTT
	reverse	CGCCCAATACGACCAAAT
RDM1	forward	TCCGGGTCTTCCCAAATGCT
	reverse	GTGCCAAGACGAACCTTGACTG
CDCA3	forward	TAACTTCGGGAGTTGAGCCAC
	reverse	CTGTTTCACCAGTGGGCTTG
FLVCR1	forward	GTAGCTGGAATGGTGGGCTC
	reverse	GAAGAAGCCAAGCACCCCTC

**Table 2:** The result of univariate survival analysis in TCGA cohort.

Variables	Value	N	Death	Median (days)	HR (95%CI)	*P*
Risk	low	171	49	2131	1.00	
	high	172	74	1397	2.01 (1.4, 2.89)	< 0.001
Cluster	cluster2	120	36	2116	1.00	
	cluster1	223	87	1694	1.62 (1.09, 2.39)	0.015
Age (years)	60	178	69	1622	1.00	
	< 60	165	54	2532	0.86 (0.6, 1.22)	0.395
Gender	female	110	49	1490	1.00	
	male	233	74	2486	0.8 (0.56, 1.15)	0.229
AFP (ng/ml)^a^	< 400	199	56	2456	1.00	
	>= 400	61	21	2486	1.1 (0.66, 1.83)	0.702
Child-pugh^b^	A	204	52	3125	1.00	
	B+C	21	9	1005	1.85 (0.91, 3.77)	0.087
MVI^c^	no	188	54	2456	1.00	
	yes	101	35	2486	1.48 (0.96, 2.27)	0.071
Pathological T^d^	T1	168	41	2456	1.00	
	T2	84	28	1852	1.53 (0.95, 2.48)	0.081
	T3	75	43	770	2.95 (1.92, 4.54)	< 0.001
	T4	13	10	558	6.7 (3.23, 13.89)	< 0.001
TNM stage^e^	I	161	37	2532	1.00	
	II	77	24	1852	1.52 (0.91, 2.54)	0.110
	III	80	45	770	3.07 (1.98, 4.75)	<0.001
Histologic grade^f^	G1	53	17	2116	1.00	
	G2	161	58	1694	1.23 (0.71, 2.11)	0.459
	G3	112	39	1622	1.19 (0.67, 2.1)	0.556
	G4	12	5	NA	2.04 (0.71, 5.81)	0.175

Clinical variables with missing values: a, b, c, d, e and f. N: number of patients; MVI: microvascular invasion; NA: not available; HR: hazard ratio; 95% CI:95% confidence interval. HR (95%CI): calculated by Cox proportional hazards regression model. P: calculated by log-rank test.

**Table 3:** The result of univariate survival analysis for recurrence in TCGA cohort.

Variables	Value	N^g^	Recurrence	Median (days)	HR (95%CI)	*P*
Risk	low	151	60	1117	1.00	
	high	150	79	491	1.78 (1.27, 2.49)	0.001
Cluster	cluster2	105	40	1453	1.00	
	cluster1	196	99	598	1.78 (1.23, 2.57)	0.002
Age (years)	60	155	74	776	1.00	
	< 60	146	65	1509	0.89 (0.64, 1.24)	0.488
Gender	female	94	45	893	1.00	
	male	207	94	875	0.98 (0.69, 1.4)	0.919
AFP (ng/ml)^a^	< 400	172	77	912	1.00	
	>= 400	51	21	1509	0.94 (0.58, 1.52)	0.788
Child-pugh^b^	A	180	83	990	1.00	
	B+C	16	8	1286	1.54 (0.74, 3.2)	0.242
MVI^c^	no	167	64	1279	1.00	
	yes	87	43	644	1.54 (1.05, 2.27)	0.028
Pathological T^d^	T1	144	47	2028	1.00	
	T2	75	38	754	2.02 (1.31, 3.1)	0.001
	T3	67	45	301	3.81 (2.52, 5.78)	< 0.001
	T4	12	7	289	5.56 (2.41, 12.83)	< 0.001
TNM stage^e^	I	138	45	1509	1.00	
	II	69	34	754	1.93 (1.23, 3.01)	0.003
	III	72	47	297	3.67 (2.42, 5.56)	< 0.001
Histologic grade^f^	G1	50	21	990	1.00	
	G2	141	64	754	1.23 (0.75, 2.02)	0.408
	G3	97	48	828	1.25 (0.75, 2.09)	0.393
	G4	8	2	NA	0.61 (0.14, 2.61)	0.500

Clinical variables with missing values: a, b, c, d, e and f. g: 42 patients lack data of recurrence. N: number of patients; MVI: microvascular invasion; NA: not available; HR: hazard ratio; 95% CI:95% confidence interval. HR (95%CI): calculated by Cox proportional hazards regression model. P: calculated by log-rank test.

**Table 4:** The result of univariate survival analysis in ICGC cohort.

Variables	Value	N	Events	Median (years)	HR (95%CI)	*P*
TMNstage	I	37	1	NA	1.00	
	II	109	18	NA	6.5 (0.87, 48.71)	0.036
	III	73	15	NA	9.28 (1.22, 70.32)	0.009
	IV	21	9	3.29	21.75 (2.74, 172.42)	< 0.001

HR: calculated by Cox proportional hazards regression model. *P*: calculated by log-rank test. NA: not available.
